# Physicochemical, Antioxidant, Starch Digestibility, and Sensory Properties of Wheat Bread Fortified with Taiwanese Cocoa Bean Shells

**DOI:** 10.3390/foods13172854

**Published:** 2024-09-08

**Authors:** Chun-Wei Wang, Hui-Shan Shen, Chih-Wei Yang, Pei-Ci Syu, Sheng-Dun Lin

**Affiliations:** Department of Food Science and Technology, Hungkuang University, Taichung 433304, Taiwan; w506016@gmail.com (C.-W.W.); roxenshen33@gmail.com (H.-S.S.); n91768oris@sunrise.hk.edu.tw (C.-W.Y.); spc679163@gmail.com (P.-C.S.)

**Keywords:** cocoa bean shells, functional bread, physicochemical quality, antioxidant property, starch hydrolysis, predicted glycemic index, sensory evaluation

## Abstract

The effects of replacing 5–25% of wheat flour (WF) with Taiwanese cocoa bean shells (CBSs) on the physicochemical, antioxidant, starch digestion, and sensory properties of the bread were studied. The lead (0.18) and cadmium (0.77) contents (mg/kg) of the CBSs were below the Codex Alimentarius specifications for cocoa powder. Ochratoxin A and aflatoxins (B_1_, B_2_, G_1_, and G_2_) were not detected in the CBSs. The CBSs were rich in dietary fiber (42.9%) and bioactive components and showed good antioxidant capacity. The ash, fat, protein, dietary fiber, crumb a* and c*, hardness, chewiness, total phenols, and antioxidant activities of the bread increased with an increasing CBSs level. The starch hydrolysis rate (45.1–36.49%) of the CBS breads at 180 min was lower than that of the control (49.6%). The predicted glycemic index of the bread (CBS20 and CBS25) with 20–25% of the WF replaced with CBSs was classified as a medium-GI food using white bread as a reference. In the nine-point hedonic test, the overall preference scores were highest for control (6.8) and CBS breads, where CBSs replaced 5–10% of WF, with scores of 7.2 and 6.7. CBS20 supplemented with an additional 20–30% water improved its volume, specific volume, and staling rate, but the overall liking score (6.5–7.2) was not significantly different from the control (*p* > 0.05). Overall, partially replacing wheat flour with CBSs in the production of baked bread can result in a new medium-GI value food containing more dietary fiber, bioactive compounds, and enhanced antioxidant capacity.

## 1. Introduction

Cocoa beans are the main raw material used to make chocolate. The total global cocoa bean production in 2021 was approximately 5.242 million tons, with approximately 77.3% of cocoa beans being produced in Africa. The top five cocoa producing countries are Côte d’Ivoire, Ghana, Ecuador, Cameroon, and Nigeria [1]. The chocolate products from cocoa fruits generate a large amount of waste. Only 10% of the total weight of cocoa fruits is used for commercialization, whereas the remaining 90% is discarded as waste or by-products [2]. The cocoa bean shells (CBSs) is a by-product of cocoa bean processing for making chocolate, accounting for approximately 12–20% of cocoa beans [3]. Most of the CBSs, after separation from cocoa beans, is considered a by-product or waste, but its nutritional content is not much different from that of cocoa beans [4]. Value-added strategies for CBSs have emerged in different areas. Its new uses in the food industry, livestock feed, or industrial applications, such as biofuels, absorbents, or composites are considered the most common [4]. CBSs is not only rich in dietary fiber (39.25–66.33%) [4], but it also contains a variety of bioactive compounds [5,6], such as polyphenols (quercetin, quercetin-3-O-glucoside, catechin, and epicatechin), methylxanthine alkaloids (caffeine, theobromine, and theophylline), and fatty acids (i.e., oleic and linoleic acids).

Wheat bread is one of the oldest and most popular food products among consumers. Traditional white wheat bread is rich in carbohydrates but lacks bioactive ingredients and dietary fiber, so it may be detrimental to human health and may lead to metabolic-syndrome-related diseases [7]. Therefore, adding functional ingredients to bakery products has become increasingly popular. Adding dietary fiber can reduce the risk of chronic diseases, as well as improve the basic nutritional function of the raw materials (i.e., providing calories) [8]. Food manufacturers are trying to produce baked goods that are rich in dietary fiber and bioactive ingredients that have higher nutritional and health value, with attractive palatability. Raw materials or by-products rich in bioactive ingredients and dietary fiber can be used to functionally enhance food, so these raw materials and by-products are often combined [9].

Food waste and food processing by-products represent a serious global problem, endangering the long-term sustainability of the food supply chain [10]. Approximately one-third of the total food produced globally each year is wasted: of the 1.5 billion tons of fruits and vegetables produced each year, an estimated 500 million tons are wasted during processing or are converted into by-products such as peels, seeds, shells, pods, and pomace [11]. These by-products can be used and converted into valuable products, such as dietary fibers, polyphenols, polysaccharides, essential oils, resins, flavor compounds, and pigments [12]. Agricultural food waste and processing by-products can be sustainably used to produce value-added products that are economically and environmentally beneficial and contribute to the sustainability and circular economy of the food industry [13].

Taiwan introduced cocoa from Indonesia in 1922 for trial cultivation. Cocoa requires a high-temperature and humid environment for growth, and cocoa can only be grown at latitudes between 20 degrees north and south. Therefore, the climate in Pingtung County is the most suitable for cocoa cultivation. In the early days, the cocoa processing method was unfamiliar, and cocoa pods cannot be eaten fresh, so cocoa remained an unpopular crop fir a long time. In recent years, Taiwan’s cocoa bean cultivation technology has improved, and cocoa beans can be grown in many areas. Therefore, cocoa yield and quality have increased each year. Although the government spares no effort in promoting the development of the cocoa industry, CBSs application development is not yet either effective or diversified. Currently, many foreign researchers have used CBSs or its extracts to study functional foods, such as CBS chocolate cake [14], CBS tea [15], and CBS-extract-enhanced extra virgin olive oil jam [16], finding that CBSs extract enhances the functionality of biscuits [17] and that CBSs and walnut oil can be used as animal fat substitutes and as healthy bioactive sources for beef burgers [18].

The cocoa planting area and production in Taiwan are increasing every year, as is the amount of CBSs produced. However, few studies have focused on products using CBSs or its extracts. As such, we used Taiwanese cocoa bean shells grown without pesticides as test materials to examine their heavy metal and mycotoxins, as well as their physicochemical quality characteristics. We replaced 5%, 10%, 15%, 20%, and 25% of the wheat flour (13.5% protein) with CBSs powder to make bread and analyzed its physicochemical quality characteristics, starch digestibility, and sensory evaluation. The bioactive components and antioxidant properties of the freeze-dried CBSs extract, extraction yield, total phenols content, and the antioxidant properties of the freeze-dried bread extracts were analyzed using an aqueous solution with 50% (*v*/*v*) ethanol as the extraction solvent. In addition, the effects of adding different amounts of water on the quality characteristics of CBS bread were studied.

## 2. Materials and Methods

### 2.1. Materials and Reagents

**Figure 1:**
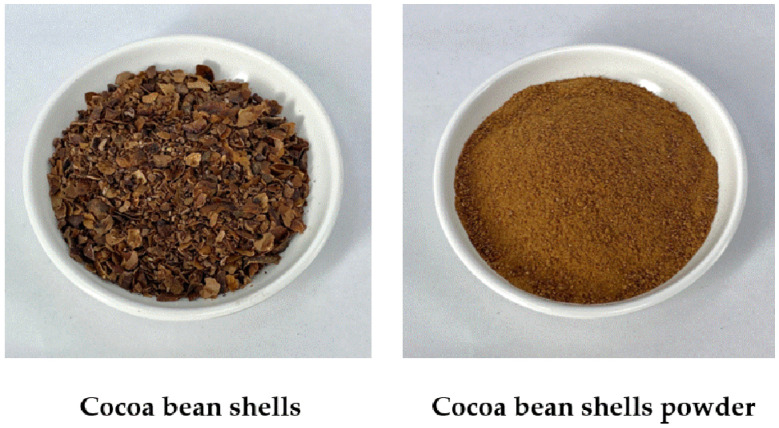
The appearance and color of cocoa bean shells and its powder.

Raw cocoa beans (Forastero variety) from plants grown without pesticides were fermented and sun-dried. We divided 32 kg of dried cocoa beans into 4 batches, and we roasted each batch in a far-infrared roller roasting machine (NTM-610, Feli Technology Co., Ltd., New Taipei, Taiwan) at 130 °C for 15 min (50 rpm). The cooled cocoa beans were poured into a cocoa bean cracker and winnower (NCM-102, Feli Technology Co., Ltd., New Taipei, Taiwan) for shelling, and the shelling yield was 12.32%. The cocoa bean shells (CBSs) (Figure 1) were ground into powder with a mill (RT-30HS, Rong Tsong Precision Technology Co., Taichung, Taiwan) and sieved (<0.7 mm). The obtained CBS powder was sealed in an aluminum foil-laminated bag (PET/Al/PE) and stored in a −25 °C freezer. Wheat flour (13.5 g protein/100 g) was purchased from Chia Fha Enterprise Co., Ltd. (Taichung, Taiwan). Sucrose (Taiwan Sugar Co., Ltd., Tainan, Taiwan), sodium chloride (Taiyen Biotech Co., Ltd., Tainan, Taiwan), Lesaffer saf-instant yeast gold (Lesaffer, Marcq-en-Baroeul, France), and unsalted butter (Fonterra brands (New Young) Pte., Ltd., Taiwan Branch, Taipei, Taiwan) were purchased from a local market.

Mycotoxin standards (aflatoxin B_1_, B_2_, G_1_, G_2_, ochratoxin A (OTA)), theobromine, caffeine, caffeic acid, catechin, epicatechin, quercetin, protocatechuic acid, gallic acid, Folin–Ciocalteu phenol reagent, ammonium formate, formic acid, 1,1-diphenyl-2-picrylhydrazyl (DPPH), potassium ferricyanide, ferric chloride hexahydrate, ferrozine, pepsin, α-amylase (heat stable), α-amylase (from porcine pancreas type VI-B), α-amylase (from human saliva, type XIII-A), 3,5-dinitrosalicylic acid, maltose, protease (from *Bacillus licheniformis*), amyloglucosidase, celite filter aid, and acetic acid were purchased from Sigma-Aldrich Chemical (St. Louis, MO, USA). Lead and cadmium were purchased from AccuStandard, Inc. (New Haven, CT, USA). Ferrous chloride tetrahydrate was purchased from Alfa Aesar (Ward Hill, MA, USA). Hexane was purchased from Tedia (Fairfield, OH, USA). Trichloroacetic acid was purchased from Thermo Scientific Chemicals Inc. (Waltham, MA, USA). Acetonitrile, methanol, and acetone were purchased from Avantor Performance Materials (Center Valley, PA, USA). Ethanol was purchased from Taiwan Tobacco & Liquor (Tainan, Taiwan). Dialysis membranes (molecular-weight cut-off of 6000–8000) were purchased from Membrane Filtration Products (Seguin, TX, USA). Cupric sulfate, anhydrous sodium carbonate, and sodium hydroxide were purchased from Shimakyu’s Pure Chemicals (Osaka, Japan); potassium chloride was purchased from Showa Chemical (Tokyo, Japan). Methylene blue and anhydrous sodium acetate were purchased from Katayama Chemical Industries (Osaka, Japan), and methyl red pure was purchased from Koch Light Research Laboratories (Gauteng, South Africa). Potassium sodium (+)-tartrate tetrahydrate was purchased from Wako Pure Chemical Industries (Osaka, Japan), and potassium sulfite was purchased from Nihon Shiyaku Reagent (Tokyo, Japan). Nitric acid (ultrapure grade, 60–70%), hydrochloric acid, sulfuric acid, sodium dihydrogen phosphate (monobasic), and sodium phosphate (dibasic, 12 hydrates) were purchased from Union Chemical (Hsinchu, Taiwan).

### 2.2. Bread Preparation

The formula (baking percentage, Table 1) and production of the control bread were based on those used in a previous study [19]. The control bread recipe involved the use of wheat flour (700 g), sugar (70 g), salt (10.5 g), yeast (7 g), unsalted butter (42 g), and water (434 g). The CBSs powder replaced 5%, 10%, 15%, 20%, and 25% (*w*/*w*) of the wheat flour in the recipe to make various breads, labeled as CBS5, CBS10, CBS15, CBS20, and CBS25, respectively. The dough was manufactured using a straight dough method. Yeast was dissolved in water at room temperature and mixed with flour, CBSs powder, sugar, and salt, using a mixer (HL-11007, San Neng Bake Ware Co., Ltd., Taichung, Taiwan) at low speed (150 rpm) for 4 min, then mixed at medium speed (316 rpm) mix for 6 min. Melted unsalted butter was added to the mixer and mixed on low speed for 4 min. After the dough was completely mixed, the dough was folded and rolled and then placed in an incubator (Chung Pu Baking Machinery, Taichung, Taiwan) and incubated at 27 °C and 75% RH for 90 min. Each piece of dough was shaped and poured into a loaf pan (196 × 106 × 110 mm, SN2052, San Neng Bakeware, Taichung, Taiwan), which was placed in an incubator at 38 °C and 85% RH for 45 min. The dough was baked in a preheated oven (K35E, Chung Pu Baking Machinery, Taichung, Taiwan) with the upper heat set at 170 °C and the bottom heat set at 230 °C for 40 min. After this, the baked bread was removed from the oven, allowed to cool to room temperature for 2 h, then removed from the baking pan and weighed. Baked breads were packed in polyethylene bags before analyzing their quality characteristics. Seven batches (two loaves of 560 g per batch) were prepared for each recipe. All treatments were randomly generated.

The effect of the use of different amounts of water on the quality characteristics of CBS20 bread was studied. The recipe for the CBS20 bread involved the use of flour (560 g), CBSs powder (140 g), sugar (70 g), salt (10.5 g), yeast (7 g), unsalted butter (42 g), and water (434 g) (Table 1). Water amounts of 5%, 10%, 15%, 20%, 25%, and 30% (*w*/*w*) were added, based on a control wheat flour amount setmat 100% (Table 1). Therefore, the total amounts of water in the CBS20W5, CBS20W10, CBS20W15, CBS20W20, CBS20W25, and CBS20W30 bread were 469 g, 504 g, 539 g, 574 g, 609 g, and 644 g, respectively.

### 2.3. Determination of Minerals

The contents of lead and cadmium in the CBSs powder were determined according to the method described by the Taiwan Food and Drug Administration [20]. Briefly, a sample (0.5 g) was placed in a microwave digestion flask, and 0.5 mL of internal standard solution (rhodium) and 6 mL of nitric acid (60–70%, ultrapure grade) were added. Then, we placed the sample in a microwave digester (SINEO Microwave Chemistry Technology, Shanghai, China) for digestion, according to the following program: 1. 1000 W, heating time 5 min, temperature 100 °C; 2. 1000 W, heating time 15 min, duration 5 min, temperature 220 °C; 3. 1800 W, heating time 10 min, duration 10 min, temperature 240 °C. After digestion and cooling, we poured the solution into a volumetric flask, washed the microwave digestion flask with 5 mL of deionized water each time, placed the washing liquid into the volumetric flask, diluted the liquid to 50 mL with deionized water, and filtered the resulting liquid through a 0.45 µm PTFE syringe filter to create the test solution. We added 0.5 mL of internal standard solution and 6 mL of nitric acid (ultrapure grade) to another blank microwave digestion flask. The subsequent steps were the same as those for the test solution to create a blank test solution. All the test solutions were analyzed using high-resolution ICP-MS (Thermo Fisher Scientific, Waltham, MA, USA). Each mineral was identified and quantified on the basis of the calibration curve of the pure compound.

### 2.4. Determination of Mycotoxins

The aflatoxin B_1_, B_2_, G_1_, G_2_, and OTA contents in the CBSs powder were measured following the method described by the Taiwan Food and Drug Administration [21]. Briefly, a sample (5 g) was placed in a centrifuge tube, and 5 mL of a phosphate buffer solution was added; the two were mixed until even. Then, 20 mL of methanol solution containing 70% acetonitrile was added, the sample was shaken for 30 min, and then centrifuged at 4300× *g* for 5 min. The supernatant of the solution (5 mL) was placed in a test tube and dried with nitrogen in a 50 °C water bath. The residue was dissolved in 20% acetonitrile solution and diluted to 1 mL, filtered through a 0.22 μm PTFE filter membrane, and used as a test solution. The test solution was analyzed with a liquid chromatography/tandem mass spectrometer (Waters Xevo TQ-MS, Milford, MA, USA) equipped with an ACQUITY BEH C18 column (2.1 mm × 10 mm, 1.7 μm; Waters Co., Milford, MA, USA). Solvent A of the mobile phase, consisting of ammonium formate (0.315 g) and formic acid (1 mL), was dissolved in deionized water to create 1000 mL, which was then filtered through a 0.22 um filter membrane. Solvent B, ammonium formate (0.315 g) and formic acid (1 mL), was dissolved in methanol to make 1000 mL, which was then filtered through a 0.22 μm PTFE filter membrane. Elution was performed with the following linear gradient: 5–85% B from 0 to 5.5 min, 85–100% B from 5.5 to 5.8 min, 100% B from 5.8 to 6.9 min, 100–5% B from 6.9 to 7.0 min, and 5–5% B from 7.0 to 9.0 min. The mobile phase flow rate was 0.3 mL/min. The sample injection volume was 10 μL, with the injection performed using an autosampler. The capillary voltage was 2.0 KV, the ion source temperature was 150 °C, ESI^+^ ionization mode was used, the desolvation temperature was 500 °C, and the desolvation flow was 1000 L/h.

### 2.5. Determination of Physical Characteristics

The water-holding capacity of the sample (g H_2_O absorbed/g sample) was measured by following a previously reported method [19]. A scale (GG4002-S, Mettler-Toledo, Langacher Greifense, Switzerland) was used to measure the weight of the bread. The bread volume was determined using the rapeseed displacement method [22]. The specific bread volume was calculated by dividing the bread volume by its weight.

The reflective color of each sample was measured with a spectrophotometer (CM-5, Konica Minolta Sensing, Inc., Tokyo, Japan), setting the CIE L*, a*, and b* values, using a D65 light source at 10° [19]. The crust color was measured on the top of the bread. The crumb was measured by cutting the midsection of the bread into cubes (3.5 cm × 3.5 cm × 3.5 cm) with a knife. The hue angle (h*) and chroma (c*) of the samples were calculated as arctan(b*/a*) and (a*^2^ + b*^2^)^1/2^, respectively. The total color difference (Δ*E*) between the sample and the control was calculated as *∆E* = [(L*_c_ − L*_s_)^2^ + (a*_c_ − a*_s_) ^2^ + (a*_c_ − a*_s_)^2^]^1/2^, where L*, a*, and b* were the color coordinates in the control (c) and CBS bread (s).

A texture analyzer (TA-XT2, 25 kg model; Stable Micro Systems, Godalming, Surrey, UK) was used to measure the texture of the middle part (2.5 cm × 2.5 cm × 2.5 cm) of the bread, according to a previous study [19]. The studied textural qualities included hardness, cohesiveness, springiness, chewiness, and resilience. Additionally, the bread hardness after being stored at room temperature for 24 h was measured, and the staling rate was calculated using the following formula [23]:

(1) $$Staling\;rate=\left(\frac{crumb\;hardness\;after\;24\;h\;\left[N\right]\;-\;crumb\;hardness\;after\;2\;h\;\left[N\right]}{hardness\;after\;2\;h\;\left[N\right]}\right)$$

### 2.6. Determination of Proximate Composition and Total Dietary Fiber

The moisture, protein, fat, and ash contents of the samples were analyzed following the methods of the American Association of Cereal Chemists [24]. The nitrogen conversion factors for the CBS powder, wheat flour, and baked bread used for crude protein calculation were 6.25, 5.7, and 5.7, respectively. The carbohydrate content (g/100 g) was 100%, minus those of moisture, protein, fat, and ash. The total dietary fiber of the sample was determined according to AOAC method 985.29 [25].

### 2.7. Determination of Bioactive Components in the Extract

We placed 15 g of powder sample into a centrifuge bottle and added 150 mL of 50% (*v*/*v*) ethanol. The mixture was then shaken in a 75 °C water bath at 150 rpm for extraction for 30 min and centrifuged (4450× *g*, 30 min). The supernatant was filtered through Advantec No. 1 filter paper. The residue was extracted again following the above steps. The two filtrates were combined, concentrated under reduced pressure (45 °C), and freeze-dried under vacuum.

The total phenols contents of the freeze-dried extracts were measured according to the Folin–Ciocalteu method [26]. To prepare the extract solution, 0.2 g of lyophilized CBSs extract or 0.5 g of lyophilized wheat flour and bread extract was weighed into a centrifuge tube, with the volume increased to 10 mL with methanol. A centrifuge tube was placed in an ultrasonic oscillator (53 kHz) for 15 min and centrifuged (1462× *g*, 10 min). The obtained supernatant was the extract solution. The total phenolic content of the extract solution were calculated following the standard curve [760 nm absorbance = 0.0009 C_gallic acid_ (μg/mL) + 0.00106, R^2^ = 0.9995]. The results are expressed as milligrams of gallic acid equivalents (GAE) per gram of lyophilized extract.

The contents of theobromine, caffeine, caffeic acid, protocatechuic acid, catechin, epicatechin, and quercetin in the lyophilized extracts were analyzed according to the method of Rojo-Poveda et al. [27]. The extract solution was prepared following the same methods used for the total phenols. The supernatant after centrifugation was filtered through a PVDF syringe filter (13 mm × 0.45 μm) and then analyzed using HPLC. The HPLC system consisted of a pump (Hitachi 5110, Hitachi High-Tech Co., Tokyo, Japan), a diode array detector (Hitachi 5430), and a Luna C18(2) column (250 mm × 4.6 mm, 5 μm particle size; Phenomenex, Torrance, CA, USA). The column temperature was maintained at 35 °C. The mobile phase consisted of water containing formic acid at 0.1% *v*/*v* (solvent A) and 100% methanol (solvent B) at a flow rate of 1.0 mL/min. Elution was performed with the following linear gradient: 10% B from 0 min to 2 min, 10–50% B from 2 to 18 min, 80% B from 18 to 40 min, 90% B from 40 to 42 min, and 10% B from 42 to 45 min. The sample injection volume was 10 μL. The bioactive components in the samples were identified by comparing their relative retention times and UV spectra with those of the verified compounds. The contents of the various bioactive components were calculated based on the calibration curves corresponding to the pure compounds.

### 2.8. Determination of Antioxidant Property of the Extract

Various concentrations of each extract were prepared in methanol (0 to 0.5 mg extract/mL for CBSs and wheat flour; 0 to 3.6 mg extract/mL for bread). The DPPH radical scavenging ability of these solutions was then determined according to the method of Shimada et al. [28]. Each extract (4 mL) was mixed with 1 mL of methanolic solution containing DPPH radicals, resulting in a final concentration of 0.2 mmol/L DPPH. The mixture was shaken vigorously and left to stand for 30 min in the dark, and the absorbance of the mixture was then measured at 517 nm against a blank.

$$\mathrm{Scavenging}\;\mathrm{ability}\;(\%)=\left\{1-\left[\frac{Absorbance\;value\;of\;sample\;added\;with\;DPPH\;-\;Absorbance\;value\;of\;sample\;added\;with\;methanol)}{\left(Absorbance\;value\;of\;control\;added\;with\;DPPH\;-\;Absorbance\;value\;of\;control\;added\;with\;methanol\right)}\right]\right\}\times 100\%$$

The reducing power of the extract solutions prepared with methanol at various concentrations (0–0.6 mg extract/mL for CBSs and wheat flour; 0–15 mg extract/mL for bread) was measured according to the method of Oyaizu [29]. Each extract (2.5 mL) was mixed with 2.5 mL of 200 mmol/L sodium phosphate buffer (pH 6.6) and 2.5 mL of 1% (*w*/*v*) potassium ferricyanide, and the mixture was incubated at 50 °C for 20 min. After adding 2.5 mL of 10% (*w*/*v*) trichloroacetic acid, the mixture was centrifuged at 200× *g* for 10 min. The upper layer (5 mL) was mixed with 5 mL of deionized water and 1 mL of 0.1% (*w*/*v*) ferric chloride, and the absorbance of the mixture was measured at 700 nm against a blank to determine the amount of ferric ferrocyanide (Prussian blue) formed. A higher absorbance indicates a higher reducing power. Reducing power = absorbance value of the sample with FeCl_3_·6H_2_O added-absorbance value of the sample without FeCl_3_·6H_2_O added.

The ferrous ion chelation ability of the extract solutions prepared with methanol at various concentrations (0–0.6 mg extract/mL for CBSs flour and wheat flour; 0–6 mg extract/mL for bread) was analyzed following the method of Dinis et al. [30]. Each extract (1 mL) was mixed with 3.7 mL of methanol and 0.1 mL of 2 mmol/L ferrous chloride. After standing for 30 s, 0.2 mL of 5 mmol/L ferrozine was added. After 10 min at room temperature, the absorbance of the mixture was determined at 562 nm against a blank. A lower absorbance indicates a higher chelating ability.

$$\mathrm{Chelating}\;\mathrm{ability}\;(\%)=\left\{1-\left[\frac{Absorbance\;value\;of\;sample\;added\;with\;reagent\;-\;Absorbance\;value\;of\;sample\;added\;with\;water)}{Absorbance\;value\;of\;control\;added\;with\;methanol}\right]\right\}\times 100\%$$

The estimated EC_50_ value (mg extract/mL) represents the effective concentration at which 50% of the DPPH free radicals are scavenged, the absorbance is 0.5 as a measure of reducing power, and 50% of the ferrous ions are chelated. The estimated EC_50_ values of the CBS and CBS bread extracts were obtained via the interpolation of the linear regression analysis, while the EC_50_ values of the wheat flour and control bread were obtained via the extrapolation of the linear regression analysis.

### 2.9. Determination of Starch Hydrolysis and Predicted Glycemic Index (pGI)

The starch hydrolysis of the baked bread (room temperature) was tested using a restricted (dialysis) system method [31]. The hydrolysis index (HI, %) was calculated by dividing the area under the hydrolysis curve of the sample by that of white bread, and the resulting value × 100%. The pGI of the starch hydrolysis value of bread at 180 min was calculated according to the following formula: pGI = 39.71 + (0.549 × HI) [32].

### 2.10. Sensory Evaluation

Sensory evaluation of the baked bread was performed using the hedonic test. Fifty untrained consumers (17 men and 33 women, aged 21–54 years) interested in the CBS breads were recruited from Hungkuang University students, staffs, and teachers. Each CBS sample (2.5 cm × 2.5 cm × 2.5 cm) was placed on a white plate, identified by a random three-digit number, and then presented to the consumer in a counterbalanced and randomized order. Consumers were asked to rinse their mouths with warm water before tasting the next sample to reduce the impact of sample residue. A nine-point hedonic scale questionnaire was used to assess the sensory quality characteristics of the bread, including appearance, crumb color, aroma, flavor, texture, and overall liking (1 = dislike very much, 5 = neither like nor dislike, 9 = like very much).

### 2.11. Statistical Analysis

Each measurement was performed four times, except for the hedonic test (*n* = 50). The variance of the experimental data was analyzed using the Statistical Analysis System (SAS Institute, Cary, NC, USA). Duncan’s multiple range test was used to determine the significance of differences among the means at the 0.05 level.

## 3. Results and Discussion

### 3.1. Heavy Metal and Mycotoxin Contents in the CBS

The heavy metal (lead and cadmium) contamination in cocoa raw materials and products has been widely studied [33,34]. The possible factors affecting the heavy metal contamination of cocoa include the use of chemical fertilizers, pesticides, and insecticides, as well as exposure to metal equipment during fermentation and drying [35]. The International Agency for Research on Cancer (IARC) lists inorganic lead compounds as Group 2A (probably carcinogenic to humans) and cadmium as Group 1 (carcinogenic to humans) [36]. However, tetraethyl lead is still commonly used in several cocoa-producing countries. During cocoa bean fermentation, drying, and transportation, emissions from the combustion of leaded gasoline may directly contact CBSs [37]. Lead poisoning involves symptoms such as anemia, weight loss, and cognitive impairment [38]. Assa et al. [39] found that the lead concentrations in CBSs range from 5.810 to 11.146 mg/kg, which are 58~111-fold higher than the lead concentrations observed in cocoa beans (0.1 mg/kg) and substantially higher than the European Union-permitted lead contamination levels of cocoa powder (1.00 mg/kg). However, these values vary widely in different studies [4]. Cadmium is easily absorbed by crops. However, even when the cumulative cadmium concentration is high, plants do not show signs of poisoning, but the concentration is enough to threaten human and animal health [40]. Cadmium affects the body’s physiological metabolism, which may be related to the development of various cancers (such as breast, kidney, pancreatic, prostate, and bladder cancers) or chronic diseases (such as Alzheimer’s disease) [41]. Lewis et al. [42] found that the cadmium content of CBSs was 0.05–0.10 mg/kg, which is lower than the Codex Alimentarius standard of 1.5 mg/kg for cocoa powder [39,43]. The Taiwan Food and Drug Administration has not yet announced the heavy metal standards for CBSs. The lead and cadmium contents (mg/kg, dry basis) in the CBSs in this study were 0.19 and 0.80 (Table 2), respectively, which are both lower than the Codex Alimentarius specification for cocoa powder (Pb < 1.0 mg/kg and Cd < 1.5 mg/kg).

The mycotoxins produced by members of the *Aspergillus* and *Penicillium* genera are also major safety concerns regarding CBSs. Cocoa beans can become contaminated during fermentation, drying, and storage. Notably, mycotoxins are heat-resistant and cannot be completely eliminated by roasting [5,44]. OTA is associated with nephrotoxic, teratogenic, and immunosuppressive activities and is classified as a 2B carcinogen (possible human carcinogen) [36,45]. The main OTA content in total cocoa beans is found in CBSs, usually accounting for approximately 50–95%, with a concentration of 0.13–2.01 g/kg, which is within the acceptable range for cocoa beans, as determined by the European Commission (<2 g/kg) [44,45,46,47]. However, the OTA of CBSs can be as high as 23.1 g/kg [48]. Aflatoxin is hepatotoxic, teratogenic, mutagenic, and carcinogenic in humans [49]. The concentrations (μg/kg) of aflatoxin B_1_, B_2_, G_1_, and G_2_ detected in the CBSs were 0.01–0.84, <0.003–0.02, <0.003–0.44, and <0.002–0.06, respectively, which are considered safe values [50]. Considering the extensive use of cocoa products by children, the Brazil National Health Surveillance Agency has set the limits for the OTA and total aflatoxin contents of cocoa beans to less than 10 and 5 µg/kg, respectively. The EU Rapid Alert System for Food and Feed (RASFF) found that the aflatoxin levels in organic cocoa powder produced in Ghana and sold in the Netherlands have increased [51]. The levels of aflatoxin B_1_, B_2_, G_1_ and G_2_, and OTA were below the detection limits in CBSs (Table 2), and the detection limits were 0.2, 0.1, 0.2, 0.1, and 0.5 µg/kg, respectively.

In summary, the CBSs used in this study as a raw food material for wheat bread should not pose any risk of food poisoning caused by lead, cadmium, and mycotoxins.

### 3.2. Physicochemical Properties of CBSs and Wheat Flour

The crude protein, fat, ash, and total dietary fiber contents of the CBSs were significantly higher than those of the WF, whereas the opposite was found for moisture and carbohydrates (Table 2). The moisture, protein, fat, ash, and dietary fiber contents (g/100 g) of the CBSs from São Tomé cocoa beans (Forastero variety) were 5.9, 20.9, 2.3, 7.9, and 55.1, respectively [27]. The moisture, protein, ash, and dietary fiber contents were higher than those in this study (Table 2), whereas the fat content was lower than that in the samples in this study. The moisture, protein, fat, ash, and total dietary fiber contents (g/100 g) of CBSs (Forastero variety) from the Republic of Ghana were 6.79–7.05, 15.59–17.13, 3.00–5.60, 7.00–7.34, and 61.18–63.14, respectively [3]. The water and dietary fiber contents were higher than those in the samples in this study, but its fat content was lower. The protein and ash contents were comparable to those of the samples in this study (Table 2). The possible reasons for the differences in the contents of proximate components of CBSs include shelling rate, production location, and cultivation and drying methods.

The water-holding capacity of the CBSs was significantly higher than that of the WF (Table 2) because the dietary fiber content of the CBSs was higher than that of the WF. The polysaccharides in dietary fiber are highly hydrophilic, and water can be retained in the hydrophilic sites of the fiber or in the interstitial spaces in the molecular structure [52].

The L* value of the WF was significantly higher than that of the CBSs, indicating that the WF was brighter than the CBSs (Table 2). The a* and b* values of CBSs are redder and yellower than those of the WF. The Δ*E* value showing the color difference between CBSs and WF was 53.49, which means that the observers would notice the differences in color between the two samples [53].

### 3.3. Bioactive Components and Antioxidant Properties of CBSs and Wheat Flour Extracts

We used 50% aqueous ethanol as the extraction solvent. The extraction yield and total phenol concentrations of the CBSs extract were significantly higher than those of the WF extract (Table 3). When the total phenolic content of the extract was converted into CBSs powder, the total phenolic content (9.06 mg GAE/g powder) was also significantly higher than that of the WF (0.70 mg GAE/g powder). The main phenolic compounds in CBSs include epicatechin, catechins, and proanthocyanidins [27,54]. The total phenolic content of CBSs from different regions of Venezuela is 5.88–7.52 g GAE/kg powder (Criollo and Trinitario) [55], which is lower than that found in this study. The possible reasons for the difference in the total phenolic content of CBSs include differences in variety, cultivation practices, drying, shelling rate, solvent, and extraction method [27,56].

Among the bioactive components in CBSs in this study, the theobromine content was the highest, followed by those of catechin > epicatechin > caffeine > caffeic acid > quercetin, whereas protocatechuic acid was not detected (Table 3). The methylxanthines in cocoa, including theobromine and caffeine, can positively affect our mood and alertness. The theobromine content of the CBSs in this study was higher than that of caffeine. This result is similar to those of Rojo-Poveda et al. [27] and Martins et al. [57]. In terms of phenolic compounds, the contents of catechin and epicatechin in the CBSs were the highest (Table 3). Cocoa polyphenol extract and epicatechin can protect human endothelial cells from oxidative damage by regulating the production of oxygen free radicals, antioxidant enzymes, and nonenzymatic defenses [57]. After the fresh cocoa beans were dried and defatted, the samples contained epicatechin concentrations of 21.89–43.27 mg/g. Fermentation reduces the epicatechin concentration of cocoa beans [58]. Rojo-Poveda et al. [27] used six home coffee-brewing techniques to extract CBSs. Methylxanthines and phenolic compounds, which are antioxidants and inhibit α-glucosidase, were detected in the extracted extracts [27].

**Figure 2:**
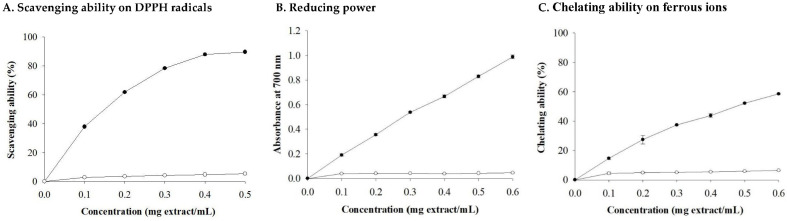
Antioxidant property of cocoa bean shells and wheat flour extracts. Each value is expressed as mean ± standard deviation (*n* = 4). CBS (●), WF (○). CBS: cocoa beans shell. WF: wheat flour.

The progression of various human diseases, such as diabetes and atherosclerosis, is related to free radicals [59]. Naturally occurring antioxidants can delay the progression of many chronic human diseases by scavenging free radicals [60]. The antioxidant capacity of the CBSs extract in this study, including its ability to scavenge DPPH radicals, reduce power, and chelate ferrous ions, was significantly enhanced with the increase in extract concentration (Figure 2). When the extract concentrations were 0.1, 0.2, and 0.4 mg extract/mL, the DPPH radical scavenging ability of the CBSs extracts was 37.89%, 61.73%, and 87.82%, respectively, whereas that of the WF extracts was only 2.88%, 3.54%, and 4.71%, respectively (Figure 2A). At a concentration of 0.1, 0.3, and 0.6 mg extract/mL, the reducing power of the CBSs extracts (0.191 AU, 0.538 AU, and 0.989 AU, respectively) was better than that of the WF extract (0.040 AU, 0.043 AU, and 0.043 AU, respectively) (Figure 2B). When the concentration (mg extract/mL) of the extract was 0.1 and 0.5, the CBSs extract had a better ability to chelate ferrous ions (14.642% and 52.273%) than the WF extract (4.566% and 5.853%) (Figure 2C). Ferrous ions can be chelated by intermolecular hydroxyl groups or adjacent hydroxyl and carbonyl groups in the molecule to form complexes [61]. Therefore, the specific functional groups in the polyphenolic structure of CBSs extracts may be one of the reasons for their ability to chelate ferrous ions. The estimated EC_50_ values (mg extract/mL) of the CBSs extract for scavenging DPPH radicals, reducing power, and chelating ferrous ions were 0.15, 0.28 and 0.47, respectively, which were substantially higher than those of the WF extract. This result is related to the fact that the CBSs extract is rich in bioactive components (Table 3).

The above results show that CBSs is a rich source of dietary fiber and bioactive components, and replacing the wheat flour in traditional wheat bread recipes with CBSs can produce functional products.

### 3.4. Color Characteristics of the Breads

**Figure 3:**
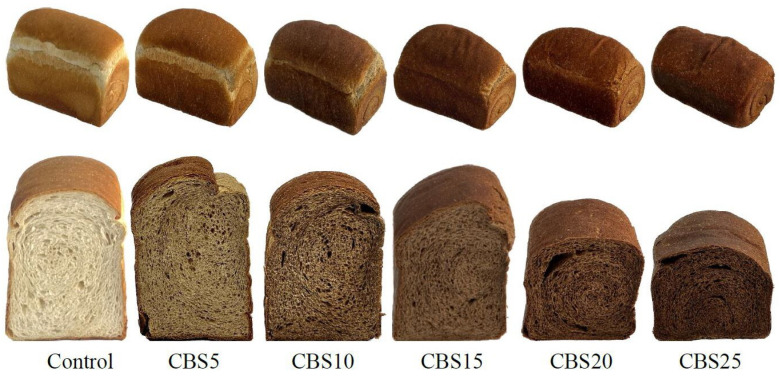
The appearance and color of cocoa bean shells breads.

Color affects the consumer acceptance of bread. Figure 3 shows the appearance, as well as the crust and crumb color of the bread made with CBSs replacing part of WF. The L*, b*, h*, and c* values of all bread crusts decreased as the amount of CBSs increased (Table 4). That is, as the amount of CBSs replacing WF increased, the color of the bread crust became darker (L*), less yellow (b*), and less saturated (c*). The h* value shows that the color of the bread crust changed from yellow to orange (59.73–47.95°) [62,63]. The Δ*E* values for CBS5, CBS10, CBS15, CBS20, and CBS25, compared to the control, were all greater than 5; the Δ*E* values for CBS10, CBS15, CBS20, and CBS25, compared to those for CBS5, were 7.72, 7.69, 8.04, and 10.5, respectively, which were also greater than 5. When the Δ*E* of two samples was greater than 5, an observer can notice two different colors [53]. The Δ*E* values for CBS15 and CBS20, compared to CBS10, were 0.03 and 0.32, respectively; the Δ*E* value for CBS20 and CBS15 was 0.35, indicating that the color difference would be unrecognizable by a standard observer. The Δ*E* values for CBS10, CBS15, and CBS20, compared to CBS25, were 2.75, 2.81, and 2.46, respectively, indicating that inexperienced observers could spot the difference [53].

The a*, b*, c*, and Δ*E* values of the crumb increased as the amount of CBSs replacing WF increased, whereas the trends in the L* and h* value were the opposite (Table 4). This shows that the redness, yellowness, and chroma of the crumb strengthened and darkened as the amount of CBSs replacing WF increased. The h* value showed that as the amount of CBSs replacing WF increased, the color of the bread crumbs changed from yellow-green to yellow (88.69–60.61°) [62,63]. Based on the Δ*E* value of the crumbs, an observer could notice two different colors between breads. The Δ*E* between CBS15 and CBS20 was 3.61, which means any observer could easily notice the difference [53]. The change in crust color of the baked bread may be related to the Maillard reaction, caramelization reaction, and the pigment of CBSs [64]. The internal temperature of the bread during the baking process does not exceed 100 °C, so the color of the crumb is usually affected by the color of the flour and the raw materials. Therefore, the crumb is less prone to the Maillard and caramelization reactions, which creates color changes [65].

### 3.5. Weight, Volume, Texture, and Proximate Composition of the Breads

The weight of the bread increased as the amount of CBSs in the bread increased (Table 5). This may have been due to the total dietary fiber content and water-holding capacity of CBSs being significantly higher than those of the WF (Table 2), making the moisture in the bread with a higher CBSs content less likely to evaporate during the baking process, as the weight of the dough put into the mold is the same (560 g). The moisture content of CBSs (4.31%) was significantly lower than that of wheat flour (13.55%) (Table 2); therefore, bread with higher CBSs content retains higher solid content. The volume and specific volume of the bread decreased as the amount of CBSs replacing the WF increased (Table 5). The reduction in bread volume was due to the effect of gluten dilution. Although the crude protein content of the bread increased as the amount of CBSs replacing the WF increased (Table 5), the protein structure of CBSs was different from that of WF (glutenin and gliadin). Therefore, as the amount of CBSs replacing WF increased, the gluten content of the bread also decreased, and its volume decreased. In addition, dietary fiber competes with gluten for water, thereby hindering the normal development of a dough’s gluten network. The dilution of the dough’s gluten changes the continuity of the gluten network, causing the gluten network structure to weaken and the dough air cells to rupture during the early fermentation process. This results in less dough expansion [66].

Hardness is often used as an indicator of bread quality. As the amount of CBSs replacing WF increased, the hardness of the bread also increased (Table 5). Increases in bread hardness are related to its volume and specific volume. The volume of bread decreased with the increase in CBSs addition (Table 5), and the hardness increased. Cohesiveness refers to the degree to which food can withstand deformation before being destroyed. This means that bread with high cohesion forms clumps rather than disintegrating during chewing [67]. The results of this study showed that when CBSs replaced up to 25% of the WF, the cohesiveness of the bread significantly decreased, which may have been caused by the decrease in gluten content. Springiness is measured as the degree of recovery between the first and second compressions. Our findings showed that when the amount of CBSs replacing WF was 20% and 25%, the springiness of bread significantly decreased. This may have been due to the gradual increase in the proportion of CBSs replacing WF in the bread formula, resulting in a lower gluten content, smaller volume, and higher density. Chewiness is calculated as hardness × cohesiveness x springiness. Chewiness represents the force required to chew food, which involves the chewing characteristics of teeth, such as the compression, shearing, cutting, tearing, and crushing of food. The results showed that when the amount of CBSs replacing WF was more than 15%, the chewiness of bread significantly increased. Resilience is the ratio of energy that can be recovered after the first compression. The results showed that when the amount of CBSs replacing WF was 20% and 25%, the resilience of bread significantly decreased (*p* < 0.05). In summary, replacing WF with CBSs increases the hardness, gumminess, and chewiness of bread but also reduces springiness, cohesiveness, and resilience. These changes are primarily attributed to gluten dilution. If the CBSs is used to replace the WF in bread, the number of air cells may be considerably reduced, resulting in a reduction in specific volume, thereby reducing springiness and increasing hardness.

The ash, fat, protein, and total dietary fiber contents of the bread increased as the amount of CBSs replacing WF increased, but the moisture and carbohydrate contents gradually decreased (Table 5). This could be attributed to the differences in proximate compositions between CBSs and WF (Table 2).

### 3.6. Starch Hydrolysis and pGI of the Breads

**Figure 4:**
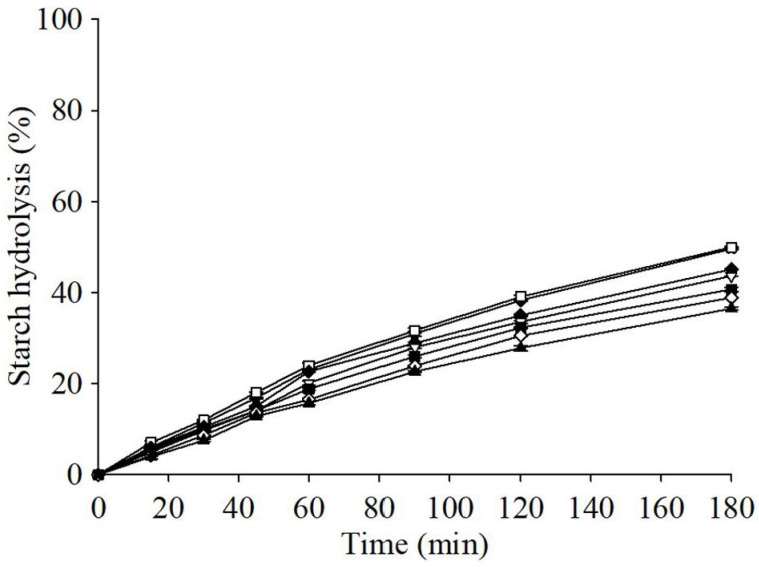
Starch hydrolysis of control and cocoa bean shells breads. Each value is expressed as mean ± standard deviation (*n* = 4). White bread (★), control (□), CBS5 (◆), CBS10 (▽), CBS15 (■), CBS20 (◇), CBS25 (▲).

Measuring the GI value of foods in vivo is a resource-intensive and relatively time-consuming experiment. The in vitro enzymatic method is used to determine the rates of starch digestion and glucose absorption in the small intestine, which is particularly suitable for screening starch-rich products during product development prior to in vivo gastrointestinal testing. Therefore, the in vitro enzymatic method is a faster and more cost-effective method [32,68]. The pGI of baked bread in this study was estimated using the in vitro enzyme hydrolysis of starch. Using white bread as a reference, the starch hydrolysis was 49.6% at 180 min in the restricted system (Figure 4). This result is similar to that in previous studies (42–50%) [31,68,69,70]. Figure 4 depicts the starch hydrolysis curve of the baked bread in this study. At the end of the 180 min incubation period, the starch hydrolysis percentages of CBS5 (45.1%), CBS10 (43.6%), CBS15 (40.7%), CBS20 (38.9%), and CBS25 (36.5%) were significantly lower than that of the control (50.0%). This result indicates that bread made by replacing part of the WF with CBSs may be more beneficial to the health of consumers and those with type 2 diabetes. When using white bread as the reference food, the pGI values of the control, CBS5, CBS10, CBS15, CBS20, and CBS25 ranged from 95.99 to 79.44 (Table 6). Among the breads tested, CBS20 and CBS25 were medium-GI foods [71], possibly due to their total dietary fiber and bioactive ingredients.

### 3.7. Hedonic Quality Characteristics of the Breads

**Figure 5:**
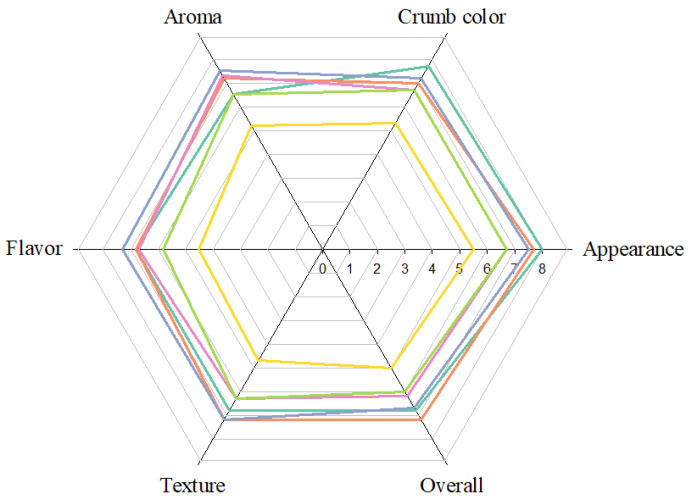
The radar plot of hedonic quality characteristic results of cocoa bean shells breads. Each value is expressed as mean (*n* = 50). Control (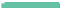), CBS5 (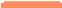), CBS10 (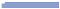), CBS15 (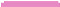), CBS20 (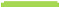), CBS25 (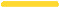). Nine-point hedonic scale with 1, 5, and 9 representing extremely dislike, neither like nor dislike, and extremely like, respectively.

The appearance and crumb color scores of control (8.0 and 7.7), CBS5 (7.7 and 7.0), and CBS10 (7.5 and 7.2) were not significantly different (*p* > 0.05) (Figure 5), and were higher than those of other samples. The control (6.5 and 6.8), CBS5 (7.2 and 6.8), CBS10 (7.5 and 7.3), and CBS15 (7.6 and 6.7) exhibited no significant differences in aroma and flavor scores, which were higher than those of other breads. The texture score of control (6.8), CBS5 (7.2), and CBS10 (7.2) was not significantly different and was higher than that of the other samples. There was no significant difference in the overall liking scores of the control (6.8), CBS5 (7.2), and CBS10 (6.7), while CBS25 (5.0) had the lowest score. Based on the data obtained, high levels of CBSs will reduce the hedonic quality characteristic scores of the bread. Changes in these hedonic quality characteristics are positively correlated with the volume and specific volume of the CBS bread and negatively correlated with hardness and chewiness. Among all the samples, the bread made with CBSs replacing 25% of WF had the lowest score.

### 3.8. Total Phenols and Antioxidant Properties of the CBS Bread Extracts

**Figure 6:**
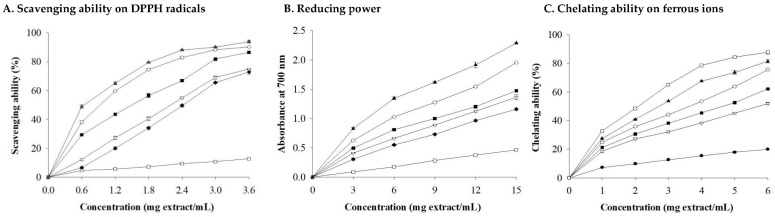
Antioxidant property of cocoa bean shells bread extracts. Each value is expressed as mean ± standard deviation (*n* = 4). Control (□), CBS5 (◆), CBS10 (▽), CBS15 (■), CBS20 (◇), CBS25 (▲).

In this study, 50% aqueous ethanol was used as the extraction solvent for the bread. The extraction yield (20.15–28.27%) and total phenolic content phenolic content (3.64–11.91 mg GAE/g lyophilized extract) of the obtained extract increased with the increase in the amount of CBS replacing WF (Table 7). This was due to the total phenolic content of the CBSs being significantly higher than that in the WF (Table 2). The antioxidant ability increased in a dose-dependent manner with the increase in bread extract concentrations (Figure 6). At a dose of 0.6 mg extract/mL, the CBS25 extract (49.05%) had the strongest ability to scavenge DPPH radicals, followed by CBS20 (38.24%), CBS15 (29.36%), CBS10 (12.29%), CBS5 (6.69%), and the control (4.71%) (Figure 6A). When the extract concentration increased to 2.4 mg extract/mL, the order of the extract’s ability to scavenge DPPH radicals was CBS25 (88.12%) > CBS20 (82.88%) > CBS15 (66.93%) > CBS10 (54.95%) > CBS5 (49.67%) > control (9.45%). At 3.6 mg extract/mL, the scavenging ability of the CBS25 and CBS20 extracts was more than 90%. The estimated EC_50_ value (mg extract/mL) showed that the ability of the bread extract to scavenge DPPH radicals in the descending order was CBS25 (0.64) > CBS20 (0.93) > CBS15 (1.50) > CBS10 (2.19) > CBS5 (2.41) > control (15.07).

When the concentration was 3 mg extract/mL, the reducing power of the extract followed the order of CBS25 (0.83AU) > CBS20 (0.63 AU) > CBS15 (0.50 AU) > CBS10 (0.41 AU) > CBS5 (0.31 AU) > control (0.09 AU) (Figure 6B). When the extract concentration was increased to 15 mg extract/mL, the reducing power of the extracts, in descending order, was CBS25 (2.29 AU) > CBS20 (1.95 AU) > CBS15 (1.47 AU) > CBS10 (1.36 AU) > CBS5 (1.16 AU) > control (0.47 AU). The estimated EC_50_ value (mg extract/mL) showed that CBS25 (1.67) had the strongest reducing power, followed by CBS20 (2.50), CBS15 (3.21), CBS10 (4.15), CBS5 (5.34), and the control (16.79).

When the dose was 1 mg extract/mL, CBS25 (32.78%) had the strongest ability to chelate ferrous ions, followed by CBS20 (27.52%) > CBS15 (24.87%) > CBS10 (21.28%) > CBS5 (18.63%) > control (7.30%) (Figure 6C). When the dose was increased to 6 mg extract/mL, the ability of the extracts to chelate ferrous ions in was, descending order, CBS25 (87.80%) > CBS20 (81.51%) > CBS15 (75.66%) > CBS10 (62.16%) > CBS5 (51.97%) > control group (19.92%). The results of the estimated EC_50_ value (mg extract/mL) showed that CBS25 (2.09) had the strongest ability to chelate ferrous ions, followed by CBS20 (2.71), CBS15 (3.63), CBS10 (4.64), CBS5 (5.69), and the control (17.54).

Based on the above results, we found a significant positive correlation between the total phenols content and the antioxidant capacity of the extract.

### 3.9. The Impact of Adding Additional Water on the Quality Characteristics of CBS20

**Figure 7:**
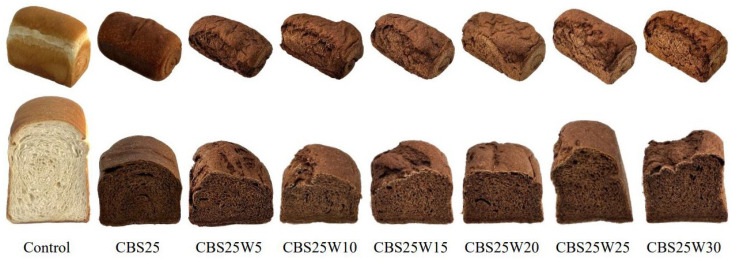
The appearance and color of control and CBS25 breads with various amounts of water added.

CBS25 had the highest content of total dietary fiber (8.12 g/100 g) (Table 5) and total phenols (11.91 mg GAE/g lyophilized extract) (Table 7), the lowest starch hydrolysis rate (Figure 4), and the strongest antioxidant ability (Figure 6). Therefore, we first explored the feasibility of adding additional water to make CBS25 bread. The results showed that the skins of all breads had large tear openings, the tissue was too solid and rough (Figure 7), and the purpose of improvement was not achieved. Because the gluten concentration of the dough was diluted, the gluten network structure weakened, and the air cells of the dough ruptured during the early fermentation process, resulting in less dough expansion [66].

**Figure 8:**
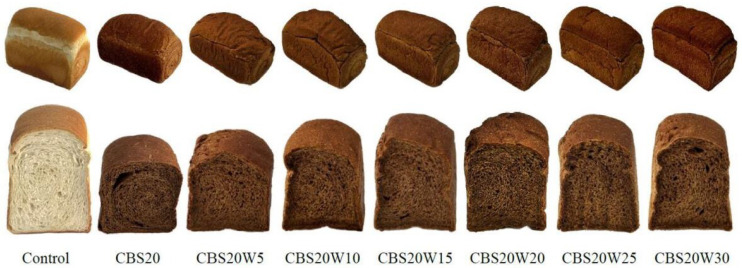
The appearance and color of control and CBS20 breads with various amounts of water added.

As such, we explored the impact of adding additional water on the quality characteristics of CBS20 bread, which also has medium GI. Adding an appropriate amount of water to CBS20 improved its appearance (Figure 8). We found no significant difference in the weights (488–498 g) of CBS20W5, CBS20W10, CBS20W15, CBS20W20, CBS20W25, and CBS20W30 (Table 8), but the weights of these breads were all significantly lower than those of the control and CBS20. This is because the weight of the dough placed in the mold was 560 g. Theoretically, the bread with less water added before baking (CB20) contained more solids than the bread with more water added before baking. Furthermore, the dough was baked at a high temperature in the oven, and the moisture in the sample with additional water added easily evaporated before the crust developed a hardened surface. The volume of the bread significantly increased with increased added water amount (Table 8), with the volume of CBS20W30 (2283 mL) bread being closest to that of the control (2342 mL). This may be caused by the yeast in the dough having enough moisture to ferment. In addition, when water turns to steam, it expands to 1100 its original volume. Steam is one of the important leavening agents [72]. Thus, the moisture in the dough will form steam during the baking process, which will also help increase the volume of the bread.

The WF in bread does not fully absorb water, which leads to a low gluten yield and restricted expansion [70]. Additionally, if dough lacks free water to maintain the normal metabolic activity of the yeast, carbon dioxide production is insufficient. In this study, by increasing the amount of water, the gluten in CBS20 bread not only fully expanded but also contributed to an increased yeast proliferation rate and gas production, so the volume of the bread also significantly increased. Specific volume is one of the most important parameters in bread making, which indicates the final gas retention of bread, affecting consumer preference [73]. We found no significant difference in the weight of bread made by adding additional water to CBS20, and we found a positive correlation between specific volume and volume. The increase in bread hardness is often used as an indicator of bread staling, which reflects the deterioration of bread quality and reduces consumer acceptability [74]. The staling rates of the samples were determined from the hardness changes 2 h and 24 h after baking (Table 8). Among all samples, CBS20W30 and CBS20W25 had the slowest staling rate, whereas CBS20 (1.67) had the fastest staling rate. This result indicated that increasing the moisture content of CBS20 slowed its staling rate. This may have occurred because the higher humidity of the more hydrated dough increased the number of disulfide bonds during mixing [75]. The above results show that adding additional water can improve the physical properties of CBS20.

The CBS20 formula with an additional 20–30% water produced the bread with the highest flavor score, with no significant difference (Table 8). There was no significant difference in texture and overall liking scores among CBS20W15, CBS20W20, CBS20W25, and CBS20W30. Among them, CBS20W25 scored the highest, significantly improving the hedonic quality characteristics of CBS20.

## 4. Conclusions

We used CBSs obtained from cocoa grown without pesticides in Taiwan as the test material. The heavy metal content of the CBSs was lower compared to the EU limits, and no mycotoxins were detected. The CBSs was found to be rich in dietary fiber and bioactive components, and the CBSs showed strong antioxidant properties. We successfully developed a functional and novel type of bread. Compared with traditional bread made with 100% WF, CBSs bread contains more dietary fiber and bioactive components, as more CBSs is used to replace WF. The incorporation of CBSs can reduce the GI value of traditional bread and provide a richer cocoa flavor. In addition, adding an additional 20–30% water to the CBS20 formula can improve its appearance, color, volume, staling rate, and hedonic quality characteristics. We think that the results of this study should serve as a reference for governments, farmers, and food processors to promote the recycling of CBSs in the cocoa bean industry and achieve the goal of zero waste.

## Figures and Tables

**Table 1:** Formulae of cocoa bean shells breads.

Ingredient (%)	Control ^1^	CBS5	CBS10	CBS15	CBS20	CBS25	CBS20W5	CBS20W10	CBS20W15	CBS20W20	CBS20W25	CBS20W30
Wheat flour	100	95	90	85	80	75	80	80	80	80	80	80
CBS powder ^1^	0	5	10	15	20	25	20	20	20	20	20	20
Sugar	10	10	10	10	10	10	10	10	10	10	10	10
Sodium chloride	1.5	1.5	1.5	1.5	1.5	1.5	1.5	1.5	1.5	1.5	1.5	1.5
Yeast	1	1	1	1	1	1	1	1	1	1	1	1
Unsalted butter	6	6	6	6	6	6	6	6	6	6	6	6
Water	62	62	62	62	62	62	67	72	77	82	87	92
Total	180.5	180.5	180.5	180.5	180.5	180.5	185.5	190.5	195.5	200.5	205.5	210.5

^1^ Control, CBS5, CBS10, CBS15, CBS20, and CBS25: bread prepared with 0%, 5%, 10%, 15%, 20%, and 25% (*w*/*w*) replacement of wheat flour with cocoa bean shells powder, respectively. CBS20W5, CBS20W10, CBS20W15, CBS20W20, CBS20W25, and CBS20W30: formula of CBS20 with an additional 5%, 10%, 15%, 20%, 25%, and 30% (*w*/*w*) water, respectively, relative to the amount of wheat flour in the control, which was set at 100%.

**Table 2:** Heavy metals, mycotoxins, and physicochemical quality characteristics of cocoa bean shells and wheat flour.

	CBSs ^1^	WF
Heavy metals (mg/kg, dry basis)		
Pb	0.19 ± 0.01 ^2^	<0.01
Cd	0.80 ± 0.02	<0.01
Mycotoxins (µg/kg, dry basis)		
Aflatoxin B_1_	<0.2	<0.2
Aflatoxin B_2_	<0.1	<0.1
Aflatoxin G_1_	<0.2	<0.2
Aflatoxin G_2_	<0.1	<0.1
Ochratoxin A	<0.5	<0.5
Proximate composition (g/100 g)		
Moisture	4.31 ± 0.30 b	13.55 ± 0.07 a
Crude protein	16.22 ± 0.50 a	13.51 ± 0.07 b
Crude fat	10.33 ± 0.33 a	1.22 ± 0.03 b
Crude ash	7.40 ± 0.33 a	0.47 ± 0.02 b
Carbohydrate	61.74 ± 0.29 b	71.25 ± 0.07 a
Total dietary fiber	42.90 ± 0.76 a	1.96 ± 0.04 b
Water holding capacity (g water absorbed/g sample)		
	3.47 ± 0.04 a	0.83 ± 0.01 b
Color property		
L*	40.35 ± 0.24 b	89.98 ± 0.10 a
a*	15.21 ± 0.20 a	0.16 ± 0.04 b
b*	23.02 ± 0.38 a	9.95 ± 0.17 b
Δ*E*		53.49 ± 0.11

^1^ CBSs: Cocoa bean shells. WF: wheat flour. ^2^ Each value is expressed as mean ± standard deviation (*n* = 4). Means with different lowercase letters within a row differ significantly (*p* < 0.05).

**Table 3:** The yield and bioactive components of cocoa bean shells powder and wheat flour extracts.

	Content (mg/g Lyophilized Extract)	
	CBSs ^1^	WF
Yield (%) ^2^	27.35 ± 0.05 a ^4^	9.99 ± 0.06 b
Total phenols ^3^	33.12 ± 0.18 a	7.04 ± 0.22 b
Theobromine	119.1 ± 0.1	<10^−5^
Caffeine	3.44 ± 0.08	<10^−5^
Epicatechin	10.05 ± 0.14	<10^−4^
Catechin	15.12 ± 1.07	<10^−4^
Caffeic acid	0.31 ± 0.04	<10^−5^
Protocatechuic acid	<10^−5^	<10^−5^
Quercetin	0.04 ± 0.01	<10^−4^

^1^ CBSs: cocoa beans shells. WF: wheat flour. ^2^ Yield (%) = (weight of lyophilized extract/weight of sample) × 100%. ^3^ Total phenols unit: mg gallic acid equivalent/g lyophilized extract. ^4^ Each value is expressed as mean ± standard deviation (*n* = 4). Means with different lowercase letters within a row differ significantly (*p* < 0.05).

**Table 4:** Color property of cocoa bean shells breads.

	Control ^1^	CBS5	CBS10	CBS15	CBS20	CBS25
Crust color property						
L*	52.31 ± 2.52 a ^2^	43.14 ± 0.79 b	34.51 ± 0.79 c	34.41 ± 0.47 c	34.30 ± 0.97 c	32.33 ± 0.51 d
a*	13.02 ± 0.75 b	13.78 ± 0.18 a	13.82 ± 0.37 a	13.93 ± 0.32 a	13.48 ± 0.33 ab	12.87 ± 0.72 b
b*	22.31 ± 0.62 a	16.89 ± 0.59 b	16.48 ± 0.93 b	16.92 ± 0.74 b	16.09 ± 0.73 b	14.27 ± 0.78 c
*h* (°)	59.73 ± 1.62 a	50.78 ± 1.25 b	49.99 ± 0.89 b	50.52 ± 0.81 b	50.04 ± 0.73 b	47.95 ± 1.16 c
c*	25.84 ± 0.65 a	21.80 ± 0.40 b	21.51 ± 0.94 b	21.92 ± 0.74 b	20.99 ± 0.76 b	19.22 ± 0.98 c
Δ*E*		10.77 ± 2.07 c	18.78 ± 2.69 b	18.75 ± 2.31 b	19.10 ± 2.55 b	21.56 ± 2.91 a
Crumb color property						
L*	78.42 ± 2.35 a	56.88 ± 2.67 b	48.55 ± 2.16 c	43.07 ± 2.13 d	39.53 ± 1.40 e	34.27 ± 1.46 f
a*	0.39 ± 0.06 f	6.23 ± 0.34 e	8.64 ± 0.30 d	9.65 ± 0.34 c	10.39 ± 0.28 b	10.67 ± 0.33 a
b*	16.95 ± 0.75 d	18.05 ± 0.61 c	18.53 ± 0.52 b	18.78 ± 0.49 ab	18.88 ± 0.45 a	18.96 ± 0.43 a
*h* (°)	88.69 ± 0.17 a	70.99 ± 0.49 b	64.99 ± 0.80 c	62.80 ± 0.59 d	61.17 ± 1.02 e	60.61 ± 0.86 f
c*	16.95 ± 0.75 e	19.10 ± 0.68 d	20.45 ± 0.53 c	21.12 ± 0.55 b	21.55 ± 0.37 a	21.76 ± 0.43 a
Δ*E*		22.37 ± 3.41 e	31.05 ± 3.48 d	36.60 ± 2.82 c	40.21 ± 2.23 b	45.38 ± 2.50 a

^1^ Control, CBS05, CBS10, CBS15, CBS20, and CBS5: bread prepared with 0%, 5%, 10%, 15%, 20%, and 25%. (*w*/*w*) replacement of wheat flour with cocoa bean shells powder, respectively. ^2^ Each value is expressed as mean ± standard deviation (*n* = 4). Means with different lowercase letters within a row differ significantly (*p* < 0.05).

**Table 5:** Weight, volume, texture, and proximate composition of cocoa bean shells breads.

	Control ^1^	CBS5	CBS10	CBS15	CBS20	CBS25
Weight (g)	496 ± 2 e	497 ± 2 e	508 ± 1 d	510 ± 2 c	516 ± 1 b	520 ± 1 a
Volume (mL)	2343 ± 13 a	2204 ± 17 b	2096 ± 15 c	1788 ± 10 d	1551 ± 8 e	1213 ± 14 f
Specific volume (mL/g)	4.72 ± 0.03 a	4.43 ± 0.05 b	4.13 ± 0.04 c	3.50 ± 0.03 d	3.01 ± 0.01 e	2.33 ± 0.03 f
Texture profile analysis						
Hardness (N)	0.98 ± 0.09 d ^2^	1.04 ± 0.09 cd	1.21 ± 0.19 bc	1.30 ± 0.16 b	1.35 ± 0.16 b	4.01 ± 0.48 a
Cohesiveness	0.83 ± 0.04 a	0.82 ± 0.06 a	0.81 ± 0.05 a	0.82 ± 0.04 a	0.80 ± 0.02 ab	0.76 ± 0.05 b
Springiness	1.00 ± 0.01 a	1.00 ± 0.02 a	1.00 ± 0.02 a	0.99 ± 0.03 ab	0.98 ± 0.02 b	0.96 ± 0.02 c
Chewiness (N)	0.81 ± 0.08 c	0.85 ± 0.10 c	0.97 ± 0.15 bc	1.05 ± 0.14 b	1.06 ± 0.12 b	2.93 ± 0.40 a
Resilience	0.37 ± 0.02 a	0.37 ± 0.07 a	0.36 ± 0.03 a	0.34 ± 0.03 ab	0.32 ± 0.02 b	0.31 ± 0.02 b
Proximate composition (g/100 g)						
Moisture	35.81 ± 0.43 a	35.55 ± 0.99 a	35.36 ± 0.43 a	35.26 ± 0.03 a	34.42 ± 0.84 b	34.23 ± 0.65 b
Crude ash	1.21 ± 0.03 f	1.45 ± 0.05 e	1.51 ± 0.08 d	1.69 ± 0.02 c	1.90 ± 0.02 b	2.09 ± 0.06 a
Crude fat	2.66 ± 0.05 f	3.55 ± 0.09 e	4.39 ± 0.23 d	5.15 ± 0.08 c	5.93 ± 0.17 b	6.63 ± 0.24 a
Crude protein	7.47 ± 0.08 f	7.67 ± 0.10 e	7.94 ± 0.05 d	8.11 ± 0.14 c	8.47 ± 0.06 b	8.70 ± 0.08 a
Carbohydrates	52.85 ± 0.42 a	51.78 ± 1.03 b	50.80 ± 0.37 c	49.79 ± 0.09 d	49.28 ± 0.76 d	48.35 ± 0.74 e
Total dietary fiber	0.70 ± 0.01 f	2.08 ± 0.12 e	3.60 ± 0.11 d	5.15 ± 0.10 c	6.70 ± 0.19 b	8.12 ± 0.12 a

^1^ Control, CBS05, CBS10, CBS15, CBS20, and CBS5: bread prepared with 0%, 5%, 10%, 15%, 20%, and 25% (*w*/*w*) replacement of wheat flour with cocoa bean shells powder, respectively. ^2^ Each value is expressed as mean ± standard deviation (*n* = 4). Means with different lowercase letters within a row differ significantly (*p* < 0.05).

**Table 6:** Predicted glycemic index (pGI) of cocoa bean shells breads in the in vitro test.

Sample	HI (%)	pGI ^2^	Category
Control ^1^	102.52 ± 0.40 A ^3^	95.99 ± 0.22 A	High
CBS5	92.28 ± 0.67 B	90.37 ± 0.37 B	High
CBS10	88.01 ± 0.31 C	88.03 ± 0.17 C	High
CBS15	83.27 ± 0.35 D	85.42 ± 0.19 D	High
CBS20	77.79 ± 0.74 E	82.42 ± 0.41 E	Medium
CBS25	72.37 ± 0.46 F	79.44 ± 0.25 F	Medium

^1^ Control, CBS05, CBS10, CBS15, CBS20, and CBS5: bread prepared with 0%, 5%, 10%, 15%, 20%, and 25% (*w*/*w*) replacement of wheat flour with cocoa bean shells powder, respectively. ^2^ GI categories, with the white bread used as reference food as follows: high GI value, GI > 85; medium-GI value, 85 > GI > 60; and low GI value, GI < 60. ^3^ Each value is expressed as mean ± SD (*n* = 4). Means with different capital letters within a column differ significantly (*p* < 0.05).

**Table 7:** Yield and total phenolic content of cocoa bean shells bread extracts.

	Control ^1^	CBS5	CBS10	CBS15	CBS20	CBS25
Yield (%) ^2^	20.15 ± 0.33 d ^4^	20.87 ± 0.06 f	23.23 ± 0.39 c	25.49 ± 0.16 b	26.13 ± 0.01 b	28.27 ± 0.56 a
Total phenols (mg GAE ^3^/g lyophilized extract)	3.64 ± 0.05 f	5.52 ± 0.08 e	7.35 ± 0.06 d	8.87 ± 0.23 c	10.67 ± 0.30 b	11.91 ± 0.11 a

^1^ Control, CBS5, CBS10, CBS15, CBS20, and CBS25: bread prepared with 0%, 5%, 10%, 15%, 20%, and 25% (*w*/*w*) replacement of wheat flour with cocoa bean shells powder, respectively. ^2^ Yield (%) = (weight of lyophilized extract/weight of sample) × 100%. ^3^ GAE: gallic acid equivalent. ^4^ Each value is expressed as mean ± standard deviation (*n* = 4). Means with different lowercase letters within a row differ significantly (*p* < 0.05).

**Table 8:** Weight, volume, staling rate, and sensory evaluation of control and CBS20 breads with various amounts of water added.

	Control ^1^	CBS20	CBS20W5	CBS20W10	CBS20W15	CBS20W20	CBS20W25	CBS20W30
Weight (g)	496 ± 2 b ^2^	516 ± 1 a	488 ± 2 c	498 ± 3 c	487 ± 4 c	487 ± 3 c	486 ± 2 c	487 ± 3 c
Volume (mL)	2342 ± 13 a	1551 ± 8 h	1888 ± 35 g	2022 ± 38 f	2121 ± 13 e	2179 ± 11 d	2229 ± 18 c	2283 ± 18 b
Specific volume (mL/g)	4.72 ± 0.03 a	3.01 ± 0.01 g	3.87 ± 0.07 f	4.15 ± 0.07 e	4.35 ± 0.05 d	4.48 ±< 0.01 c	4.58 ± 0.02 b	4.71 ± 0.03 a
Texture profile analysis 2 h after baking								
Hardness (N)	0.98 ± 0.09 b	1.35 ± 0.16 a	0.96 ± 0.12 b	0.87 ± 0.10 c	0.83 ± 0.15 d	0.75 ± 0.16 e	0.69 ± 0.08 f	0.56 ± 0.10 g
Texture profile analysis 24 h after baking								
Hardness (N)	1.33 ± 0.12 c	3.57 ± 0.18 a	2.01 ± 0.16 b	1.42 ± 0.15 c	1.17 ± 0.15 d	0.96 ± 0.16 e	0.84 ± 0.09 f	0.66 ± 0.10 g
Staling rate	0.37 ± 0.19 e	1.67 ± 0.28 a	1.10 ± 0.20 b	0.63 ± 0.09 c	0.41 ± 0.09 d	0.29 ± 0.07 f	0.22 ± 0.06 g	0.18 ± 0.04 h
Hedonic quality characteristics								
Flavor	6.8 ± 0.8 a	5.8 ± 0.8 b	4.7 ± 0.5 c	5.0 ± 0.6 c	5.8 ± 0.4 b	6.3 ± 0.5 ab	6.5 ± 0.5 ab	6.3 ± 0.5 ab
Texture	6.8 ± 1.0 ab	6.3 ± 1.2 b	6.3 ± 0.5 b	6.3 ± 0.5 b	6.5 ± 0.5 ab	6.8 ± 0.4 ab	7.3 ± 0.5 a	6.5 ± 0.5 ab
Overall	6.8 ± 1.2 ab	6.0 ± 0.6 b	6.2 ± 0.4 b	6.2 ± 0.8 b	6.3 ± 0.8 ab	6.5 ± 0.5 ab	7.2 ± 0.4 a	6.5 ± 0.8 ab

^1^ Control and CBS20: bread prepared with 0% and 20% (*w*/*w*) replacement of wheat flour with cocoa bean shells powder, respectively. CBS20W5, CBS20W10, CBS20W15, CBS20W20, CBS20W25, and CBS20W30 refer to the CBS20 formula with an additional 5%, 10%, 15%, 20%, 25%, and 30% (*w*/*w*) water, respectively. ^2^ Each value is expressed as mean ± standard deviation (*n* = 4; *n* = 50 for hedonic quality characteristics). Means with different lowercase letters within a row differ significantly (*p* < 0.05). Nine-point hedonic scale with 1, 5, and 9 representing extremely dislike, neither like nor dislike, and extremely like, respectively.

## Data Availability

The original contributions presented in the study are included in the article, further inquiries can be directed to the corresponding author.
